# La fibrose rétropéritonéale: à propos de 12 cas

**DOI:** 10.11604/pamj.2017.28.194.10092

**Published:** 2017-11-01

**Authors:** Aziz El Majdoub, Abdelhak Khallouk, Moulay Hassan Farih

**Affiliations:** 1 Service d’Urologie, CHU Hassan II, Fès, Maroc

**Keywords:** Fibrose rétropéritonéale, insuffisance rénale, TDM abdominale, sondes urétérales en double j, corticothérapie, Retroperitoneal fibrosis, renal failure, abdominal computed tomography scan, double-J ureteral catheter, corticosteroid therapy

## Abstract

La fibrose rétropéritonéale (FRP) est une maladie rare. Elle se caractérise par la transformation progressive du tissu adipeux rétopéritonéal en une masse fibreuse qui enserre l'aorte, la veine cave inférieure et les voies urinaires responsable d'une altération progressive de la fonction rénale. Le mode habituel de présentation de cette maladie comporte l'association de douleurs lombaires, d'une insuffisance rénale, et d'un syndrome inflammatoire biologique. Nous rapportons 12 cas de fibrose rétropéritonéale dont nous précisons les particularités cliniques, radiologiques et thérapeutiques. Il s'agit d'une étude rétrospective portant sur douze cas de fibrose rétropéritonéale colligés au service d'urologie au CHU Hassan II de Fès durant une période de 9 ans (2005-2013). Il s'agissait de dix hommes et deux femmes. La symptomatologie clinique était très variable, dominée par la douleur lombaire qui était présente chez tous les malades et une hydrocèle chez un patient. Les explorations biologiques avaient montré une insuffisance rénale chez tous les malades et un syndrome inflammatoire chez dix patients. Le diagnostic de la maladie était suspecté dans tous les cas sur les données de l'échographie qui a montré une obstruction de la voie excrétrice supérieure sans obstacle visible chez tous les malades, et confirmé par la TDM abdominale sans injection du produit de contraste qui objectivait une lésion tissulaire rétropéritonéale engainant les vaisseaux et les voies urinaires. Dans notre série, la fibrose rétropéritonéale était idiopathique dans neuf cas. Elle était péri anévrysmale chez deux malades, et post radiothérapie chez un malade. Tous nos patients avaient bénéficié d'un drainage urinaire par sonde urétérale double J. Sept malades avaient reçu une corticothérapie. Une amélioration clinique et biologique, avec disparition de la douleur et amélioration de l'état général, a été observée chez 6 patients. A travers cette étude nous avons confirmé la rareté de la fibrose rétropéritonéale, la difficulté de son diagnostic, la fréquence de la douleur, du syndrome inflammatoire et de l'insuffisance rénale. La TDM abdominale sans injection du produit de contraste confirme le diagnostic. Le drainage urinaire est indispensable dans la plupart des cas et le suivi régulier des malades est nécessaire.

## Abstract (en)

Retroperitoneal fibrosis (RPF) is rare. It is characterized by the gradual transformation of retroperitoneal adipose tissue into a fibrous mass surrounding the aorta, the inferior vena cava and the urinary tract responsible for progressive alteration in the renal function. It usually manifests as lumbar pain associated with renal failure and biologic inflammatory syndrome. We report 12 cases of retroperitoneal fibrosis in order to determine its clinical, radiological and therapeutic features. We conducted a retrospective study of 12 patients with retroperitoneal fibrosis whose medical data were collected in the Departement of Urology at the University Hospital Hassan II, Fes over a period of 9 years (2005-2013). The study involved ten men and two women. Clinical symptomatology was very variable, dominated by lumbar pain in all patients and hydrocele in 1 patient. Laboratory tests showed renal failure in all patients and inflammatory syndrome in 10 patients. The diagnosis of retroperitoneal fibrosis was suspected in all patients on ultrasound showing obstruction of the upper urinary tract without visible obstacle. The diagnosis was confirmed by abdominal CT scan without contrast that objectified a retroperitoneal tissue lesion enveloping the vessels and the urinary tract. Nine patients had idiopathic retroperitoneal fibrosis. Two patients had perianeurysmal fibrosis and one patient had post-radiation fibrosis. All patients underwent urinary drainage by double J. ureteral catheter. Seven patients underwent corticosteroid therapy. Six patients had an improvement of clinical and laboratory test outcome with disappearance of pain and improvement of the general condition. This study confirms the rarity of retroperitoneal fibrosis, the difficulty of diagnosis, pain associated with inflammatory syndrome and renal failure as common manifestations. Abdominal CT scan without contrast is the gold standard for definitive diagnosis. Urinary drainage is essential in most cases as well as regular monitoring of patients.

## Introduction

La fibrose rétropéritonéale (FRP) est une maladie rare. Sa première description remonte à Albarran, en 1905 (péri-urétérite sténosante) mais elle ne devient une entité anatomo-clinique qu'avec ORMOND en 1948 [1]. Elle se caractérise par la présence d'une réaction fibreuse au niveau des tissus rétropéritonéaux s'organisant en une plaque rétractile. Ainsi, elle peut engainer tous les organes rétropéritonéaux et particulièrement les uretères entrainant une insuffisance rénale. L'étiologie est souvent non déterminée, on parle alors de la forme idiopathique. TDM reste le moyen d'exploration de référence pour le diagnostic, l'extension et la surveillance de cette affection. Le traitement de la fibrose rétropéritonéale est longtemps resté strictement chirurgical. Plus récemment, d'autres moyens thérapeutiques ont été proposés en s'appuyant sur des données physiopathologiques. Ainsi, l'utilisation de médicaments immunosuppresseurs, dont les corticoïdes en premier lieu, a été proposée. Nous rapportons 12 cas de FRP colligées au service d'urologie et de néphrologie de CHU Hassan II de Fès. A travers cette étude rétrospective, nous allons étudier les aspects cliniques, biologiques et radiologiques, et nous insisterons sur les aspects thérapeutiques de cette affection.

## Méthodes

Il s'agit d'une étude rétrospective reposant sur l'exploitation des dossiers de 12 patients pris en charge au service d'urologie et de néphrologie de CHU Hassan II de Fès. Ces patients ont fait l'objet d'un examen clinique minutieux et d'examens de laboratoire de routine (ECBU-NFS-Ionogramme-Vitesse de sédimentation-Fonction rénale). Les investigations radiologiques réalisées sont: l'échographie rénale, la tomodensitométrie abdominale dans tous les cas et l'urographie intraveineuse lorsque la fonction rénale est normale ou devenue normale après néphrostomie ou montée de sondes urétérales.

## Résultats

**Figure 1:**
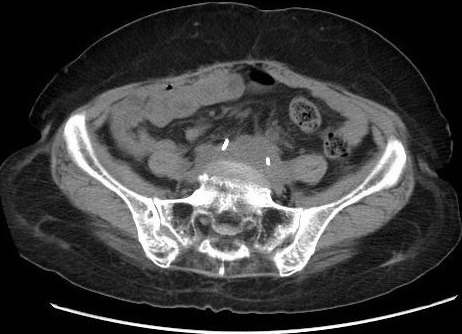
Coupe scannographique, sans injection de produit de contraste, montant une masse tissulaire rétropéritonéale, engainant les gros vaisseaux et les deux uretères, qui sont attirés vers la ligne médiane, avec des sondes urétérales double J en place

**Figure 2:**
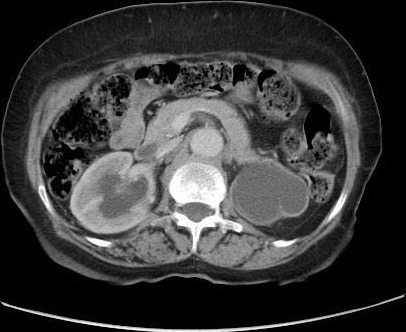
Coupe scannographique, sans injection de produit de contraste, montrant une masse tissulaire rétropéritonéale en avant du corps vertébral de la cinquième vertèbre lombaire, engainant les gros vaisseaux et les deux uretères, et responsable d’une urétèro-hydronéphrose bilatérale plus importante à gauche avec un parenchyme aminci

**Figure 3:**
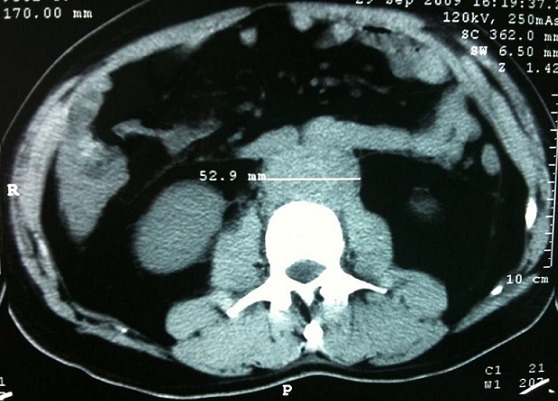
Coupe scannographique, sans injection de produit de contraste, montrant masse tissulaire rétropéritonéale mesurant 52.9 mm d’épaisseur englobant l’aorte, et les deux uretères

**Figure 4:**
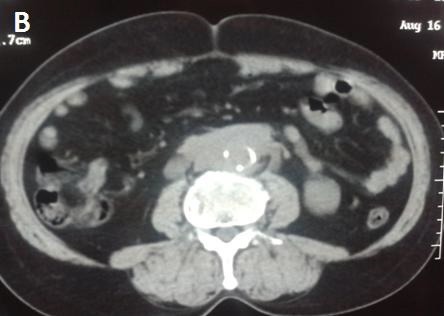
Coupe scannographique, sans injection du produit de contraste, montrant une masse tissulaire rétropéritonéale engainant les gros vaisseaux et les deux uretères avec des calcifications aortiques

**Figure 5:**
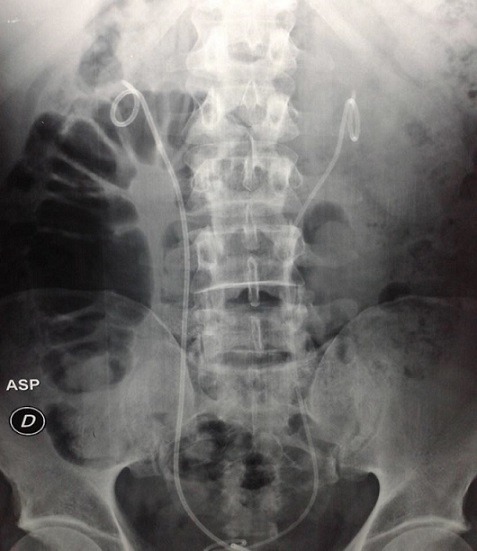
Arbre urinaire sans préparation montrant les sondes JJ en place avec attraction des deux uretères vers la ligne médiane

Il s'agissait de dix hommes et deux femmes. L'âge moyen au moment du diagnostic était de 52 ans avec des extrêmes allant de 27 à 70 ans. Une patiente avait un antécédent de cancer du col utérin traité par radiothérapie. La symptomatologie clinique était très variable, dominée par la douleur lombaire qui était présente chez tous les malades et une hydrocèle chez un patient. La majorité des patients (58,3%) inclus dans notre étude présentaient un amaigrissement non chiffré au moment du diagnostic. Les signes urinaires et les autres signes étaient variables et non significatifs et il n'y avait aucun signe clinique en faveur d'une maladie systémique ou immunitaire. L'examen clinique a retrouvé chez La plupart de nos patients une sensibilité des flancs ou des fosses lombaires à l'examen, avec des œdèmes des membres inférieurs chez deux patients, et une hydrocèle chez un malade. Les explorations biologiques avaient montré une insuffisance rénale chez 11 malades avec une moyenne de créatininémie à 90 mg/l (11-353 mg/l), et la présence d'un syndrome inflammatoire biologique .la vitesse de sédimentation était accélérée (moyenne à 75,85 mm la première heure) et une anémie normochrome normocytaire chez sept malades. Le diagnostic de la maladie était suspecté dans tous les cas sur les données de l'échographie qui a montré une obstruction de la voie excrétrice supérieure sans obstacle visible chez tous les malades et bilatérale chez 11 cas, et confirmé par la TDM abdominale qui objectivait une lésion tissulaire rétropéritonéale engainant les vaisseaux et les voies urinaires avec une épaisseur allant de 22 mm à 52 mm avec un rein détruit chez deux malades (Figure 1, Figure 2, Figure 3,Figure 4). La biopsie tissulaire avait été réalisée chez cinq malades, mettant en évidence une fibrose dense du tissu conjonctif sans aucun signe de malignité. L'UIV a montré une attraction des uretères vers la ligne médiane au niveau lombo-iliaque dans 3 cas. Dans notre série, la FRP était idiopathique dans neuf cas. Elle était péri anévrysmale chez deux malades, et post radiothérapie chez un malade. Les biopsies scanno-guidées réalisées dans 8 cas ont montré un tissu inflammatoire. Tous nos patients avaient bénéficié d'un drainage urinaire par sonde urétérale double J (Figure 5). Sept malades avaient reçu une corticothérapie associée. Une amélioration clinique et biologique, avec disparition de la douleur et amélioration de l'état général, a été observée chez 6 patients. Sur le plan biologique on a observé une amélioration du bilan inflammatoire avec normalisation de la fonction rénale chez ces patients. 4 patients ont évolué vers une insuffisance rénale persistante. Un patient est décédé au cours de son hospitalisation par Accident vasculaire cérébrale ischémique avec engagement cérébral. Un patient est perdu de vue. Aucune complication secondaire à la corticothérapie n'a été observée chez les patients au cours du suivi. Deux patients avaient un rein non fonctionnel, et ils ont bénéficié d'une néphrectomie (Tableau 1, Tableau 1 (Suite), Tableau 1 (Suite 1), Tableau 1 (Suite 2)).

**Tableau 1:** Tableau récapitulatif des observations médicales

Age/sexe	Motif de consultation	Antécédents	clinique	biologie	imagerie	Histologie	Traitement	évolution
**50/M**								
	Lombalgie droite	absents	AEG	VS accéléré	Echographie et UIV : UHN bilatérale	Prolifération fibreuse	Sondes doubles j et corticothérapie	Amélioration clinique et biologique
					TDM : plaque de FRP de 20 mm			
				insuffisance rénale				
				anémie				
**65 /F**								
	Lombalgies bilatérales	Tumeur du col utérin, radiothérapie externe et	Sensibilité des deux flans et contact	VS accélérée et anémie	Echographie : UHN bilatérale	Prolifération inflammatoire	Sondes urétérales double j et corticothérapie au début puis	Amélioration clinique et biologique
		curiethérapie	lombaire gauche		TDM : plaque de FRP de 34 mm		néphrectomie	
					UIV et scintigraphie			
					au DMSA : rein gauche muet		gauche	
**45/H**								
	Lombalgies bilatérales	HTA (inhibiteurs calciques)	AEG et sensibilité des deux	VS accéléré	-Echographie: UHN bilatérale	FRP sans signes de malignité	Sondes urétérales double j et corticothérapie	Régression de la plaque de fibrose
			flancs	insuffisance rénale	TDM : plaque de FRP de 53,2 mm et UHN			
				anémie				
**37 /H**								
	Lombalgies chroniques		hydrocèle -œdème des MI -AEG	insuffisance rénale et CRP augmentée	-écho et TDM : plaque de 50 mm péri-anévrysmale aortique et UHN		Sondes double j et corticothérapie	Amélioration clinique et biologique avec normalisation de la fonction rénale et régression de la plaque (30 mm) à 4 mois, rechute après 7 mois et évolution vers l IRC

**Tableau 1 (suite):** Tableau récapitulatif des observations médicales

Age/sexe	Motif de consultation	Antécédents	clinique	biologie	imagerie	Histologie	Traitement	évolution
			-oligurie					
**50/H**								
	Lombalgies chroniques	-tabagisme	-AEG	-VS accélérée	-écho et scanner: plaque de FRP et UHN	FRP sans signes de malignité	Montée de sonde double bilatérale et corticothérapie	Insuffisance rénale chronique
		-sciatalgie	sensibilité des deux flancs	insuffisance rénale				
				anémie				
**70/H**								
	Anurie de 48 ans	Lombalgies chroniques	HTA	VS accélérée	-écho et scanner : plaque de FRP et UHN		Montée de sonde double bilatérale et corticothérapie	-amélioration initiale de la fonction
				insuffisance rénale				-patient perdu de vue
**63/H**								
	Lombalgies chroniques avec OMI	HTA	-HTA -AEG -OMI -sensibilité des deux flancs	-insuffisance rénale - CRP augmentée -anémie hypochrome microcytaire	Echo et scanner : UHN bilatérale modérée sur masse rétropéritonéale.		Monté de sonde double j bilatérale	-après une amélioration initiale de la fonction rénale, le patient décédé au cours de son hospitalisation par AVC ischémique avec engagement cérébral.
**70/H**								
	Lombalgies chroniques	Tabagisme chronique	-AEG -sensibilité des deux fosses lombaires	-insuffisance rénale - VS accélérée -CRP augmentée	-Echo: UHN bilatérale -UPR: sténose urétérale bilatérale avec attraction vers la ligne médiane des deux uretères. TDM : plaque fibreuse rétropéritonéale péri-anévrysmale		Montée de sonde double j bilatérale	-Normalisation initiale de la fonction rénale. Bonne évolution à 6 mois de suivi avec une amélioration clinique et biologique. Patient perdu de vue depuis quelques années

**Tableau 1 (suite 1):** Tableau récapitulatif des observations médicales

Age/sexe	Motif de consultation	Antécédents	clinique	biologie	imagerie	Histologie	Traitement	évolution
**38/H**								
	Lombalgies chroniques		Sensibilité des deux flancs	-VS accélérée -insuffisance rénale	-Écho: UHN bilatérale modérée		Montée bilatérale de sondes double J.	-Amélioration clinique.
				-anémie hypochrome microcytaire	-TDM c-: masse rétropéritonéale responsable d’une UHN de moyenne abondance plus accentuée du côté gauche			-Évolution vers une IRC.
**67/F**								
	Lombalgies chroniques	-HTA sous amlodipine 10 mg -vésicule biliaire multi lithiasique		Insuffisance rénale		-Echo : UHN bilatérale laminant le parenchyme rénal par endroit. -TDM c- : UHN bilatéral en amont d’une masse rétropéritonéale avec les deux uretères qui sont attirés vers la ligne médiane.	Montée de sondes double J bilatérale.	Amélioration initiale de la fonction rénale sans normalisation

**Tableau 1 (suite 2):** Tableau récapitulatif des observations médicales

Age/sexe	Motif de consultation	Antécédents	clinique	biologie	imagerie	Histologie	Traitement	évolution
**27/H**								
	-Douleurs abdominales et lombaires chroniques		-AEG avec amaigrissement non chiffré. -Sensibilité de la fosse lombaire droite	Insuffisance rénale CRP augmentée	-Echo: rein droit de petite taille, rein gauche siège d’une UHN. -UIV : rein muet du côté droit et attraction vers la ligne médiane des deux uretères. -TDM : UHN avec atrophie rénale droite en amont d’une plaque de fibrose rétropéritonéale. -scintigraphie au DMSA/ rein muet droit.	- examen extemporané : tissu fibreux sans signes de malignité.	-Montée d’une sonde double J à gauche+ corticothérapie. -néphrectomie droite.	Amélioration clinique et biologique avec normalisation de la fonction rénale.
**51/H**								
	Lombalgies chroniques	Notion de rétention aiguë des urines. -patient sous traitement alpha bloquant.	-Sensibilité bilatérale des deux flancs. Prostate hétérogène indurée	-Insuffisance rénale. -hyperkaliémie	-Écho : UHN bilatérale modérée. -TDM : UHN bilatérale en amont d’une masse rétropéritonéale se rehaussant très faiblement au produit de contraste.		-Montée de sondes JJ. corticothérapie.	-Amélioration clinique et biologique avec normalisation de la fonction rénale.

## Discussion

La FRP est une maladie rare. Sa première description remonte à ALBARRAN, en 1905 (péri-urétérite sténosante) mais elle ne devient une entité anatomo-clinique qu'avec ORMOND en 1948 [1]. Elle se caractérise par la transformation progressive du tissu adipeux rétropéritonéal en une masse fibreuse qui enserre l'aorte abdominale, la veine cave inférieure et les uretères. Cette masse est en règle générale limitée entre le pédicule rénal en haut et le promontoire sacré en bas. Cependant, des extensions vers la grande cavité péritonéale, le médiastin et le pelvis en particulier ont été rapportées. Elle touche essentiellement l'homme âgé de 40 à 60 ans avec un âge moyen à 56 ans et une prédominance masculine [2,3]. L'âge moyen de nos patients est relativement jeune (52 ans).C'est une affection dont la physiopathologie reste inconnue, plusieurs hypothèses ont été proposées, celle d'une origine auto-immune reste la plus admise. À côté des formes idiopathiques qui représentent 70% de l'ensemble des FRP [4], il existe des fibroses secondaires : à une prise médicamenteuse (méthysergide,pergolide,bétabloquants,hydralazine, les emphétamines, haloperidol, reserpine…), à une agression rétropéritonéale (traumatisme, radiothéraie, chirurgie…) ou anévrysme inflammatoire de l'aorte abdominale. La FRP peut être dû également à un processus malin dans10% des cas (colon, rectum, estomac, sein, prostate), et seule la biopsie pourra différencier entre les FRP bénignes et FRP malignes.la FRP est parfois associé et contemporaine de certaines maladies inflammatoires comme la cholangite sclérosante, la pancréatite sclérosante, la thyroidite de riedel, une pseudotumeur rétrobulbaire et une fibrose médiastinale. Dans notre série, la FRP était idiopathique dans neuf cas. Elle était péri anévrysmale chez deux malades, et post radiothérapie chez un malade.

Le tableau clinique associe des douleurs lombo-abdominales, parfois associées à des œdèmes des membres inférieurs, une claudication intermittente, une thrombophlébite, des symptômes digestifs (nausées, vomissements), et une altération de l'état général avec un amaigrissement souvent important. Il n'y aucun signe pathognomonique de cette maladie avant de devenir compressive. Sur le plan biologique, cette affection est caractérisée par un syndrome inflammatoire avec une accélération de la vitesse de sédimentation, une anémie inflammatoire et une insuffisance rénale qui peut être notée dans les formes évoluées. L'échographie montre souvent un retentissement sur le haut appareil urinaire (dilatation urétéro-pyélocalicielle souvent bilatérale). L'urographie intra veineuse, en l'absence d'insuffisance rénale, et l'urétéro-pyélographie rétrograde qui présente la première étape du traitement peuvent poser le diagnostic de la FRP en montrant des signes caractéristiques tel que l'urétéro-hydronéphrose uni ou bilatérale, l'attraction des uretères vers la ligne médiane et la compression extrinsèques des uretères [5]. Le scanner abdomino-pelvien met en évidence la plaque, son étendue, ses limites, son retentissement sur le haut appareil urinaire et ses rapports avec les uretères explore la cavité abdominale et le rétropéritoine à la recherche d'une cause associée à la plaque de FRP, guide les biopsies afin d'éliminer un processus malin et permet aussi le suivi de l'évolution de la plaque de fibrose. Le scanner a montré chez tous nos patients les plaques de la FRP. Récemment, l'IRM a été proposée comme étant un moyen non invasif pour différencier entre la FRP bénigne et maligne [6, 7].

Le traitement de la FRPB peut faire appel à la corticothérapie qui est le choix thérapeutique de première intention. La dose des corticoïdes est de 0,5 mg/kg/j par voie orale en réservant une posologie supérieure à 1 mg/kg/j aux patients les plus graves, très fébriles ou endoloris. Certains auteurs ont proposé une corticothérapie en bolus de 500 mg à 1g pendant 3 jours relayés par des corticoïdes par voie orale. La durée du traitement est souvent en fonction de l'évolution de la maladie. Certains auteurs [8, 9] ont montré l'efficacité du tamoxifène dans le traitement de la FRP qui présente des similitudes avec les tumeurs desmoides. En cas d'insuffisance rénale obstructive, le traitement repose sur la dérivation urinaire que ce soit par néphrostomie ou par cathétérisme urétéral. Dans notre étude, une montée de sondes urétérales a été réalisée chez tous les malades. Le traitement chirurgical par urétérolyse est de moins en moins utilisé devant les bénéfices qu'apporte le drainage des voies excrétrices par sondes JJ associé ou non à la corticothérapie. L'urétérolyse avec intra-péritonisation des uretères reste la méthode la plus utilisée et celle qui protège le mieux contre les récidives. La régression spontanée de la FRP, sans recours à d'autres moyens thérapeutiques, peut survenir après cure des anévrysmes de l'aorte [10]. Ce traitement peut être proposé dans les FRP malignes. Les récidives sont très fréquentes dans les 10 premières années de l'évolution de la maladie, ce qui implique une surveillance très prolongée des patients après traitement. Cette surveillance est clinique, biologique et radiologique. Le pronostic des FRP bénignes dépend de la précocité du diagnostic et de la fonction rénale. Quant aux formes malignes leur pronostic reste effroyable. Les deux facteurs essentiels de la mortalité sont : l'âge avancé des patients; L'importance de l'insuffisance rénale au moment du diagnostic.

## Conclusion

A travers cette étude nous avons confirmé la rareté de la fibrose rétropéritonéale, la difficulté de son diagnostic qui repose essentiellement sur l'imagerie, la fréquence de la douleur, du syndrome inflammatoire et de l'insuffisance rénale. Le drainage urinaire est indispensable dans la plupart des cas. La corticothérapie occupe une place importante dans son traitement. L'observance du traitement ainsi que la surveillance des patients sont nécessaires afin d'éviter les rechutes.

### Etat des connaissances actuelle sur le sujet

- La fibrose rétropéritonéale est une maladie rare;
- Elle se caractérise par caractérise par la transformation progressive du tissu adipeux rétopéritonéal en une masse fibreuse qui enserre l'aorte, la veine cave inférieure et les voies urinaires responsable d'une altération progressive de la fonction rénale;
- La corticothérapie occupe une place importante dans son traitement.

### Contribution de notre étude à la connaissance

- Apporter notre expérience à travers cette série de cas dans la prise en charge de la fibrose rétropéritonéale;
- Confirmer la rareté de la fibrose rétropéritonéale, la difficulté de son diagnostic qui repose essentiellement sur l'imagerie, la fréquence de la douleur, du syndrome inflammatoire et de l'insuffisance rénale;
- Le drainage urinaire est indispensable dans la plupart des cas.

## Conflits d’intérêts

Les auteurs ne déclarent aucun conflit d'intérêts.

## References

[1] Gattegno B, Haab F (1992). fibrose rétropéritonéale. Encycl Med Chir6 (Editions Scientifiques et Medicale Elsevier SAS, Paris) Néphrologie-Urologue..

[2] Hartman D (1994). Retroperitoneal fibrosis. AJR..

[3] Gary S, Gilkeson MD, Nancy B, Allen MD (1996). Retroperitoneal fibrosis: a true connective disease. Rheumatic Disease Clinics of North America..

[4] Demko TM, Diamond JR, Groff J (1997). Obstructive nephropathy as a result of retroperitoneal fibrosis: a review of its pathogenesis and associations. J Am Society of Nephrology..

[5] Witten DM, Pollack HM (1990). Retroperitoneal fibrosis: clinical urography.

[6] Arrive L, Hricak H, Tavares NJ, Miller TR (1989). Malignant versus non-malignant retroperitoneal fibrosis: differenciation with MR imaging. Radiology..

[7] Brooks AP, Reznek RH, Webb JAW (1990). Magnetic resonance imaging of idiopathic retroperitoneal fibrosis: measurement of T1 relaxation time. Br J Radiol..

[8] Benson JR, Baum M (1993). Tamoxifen for retroperitoneal fibrosis. Lancet..

[9] Clark CP, Vanderpool D, Preskitt JT (1991). The response of idiopathic retroperitoneal fibrosis to tamoxifen. Surgery..

[10] Speziale F, Sbarigia E, Grossi R (2001). Inflammatory aneurysms of the abdominal aorta involving the ureters: is combined treatment really necessary?. J Urol..

